# Intussusception of Small Intestine of Colt; Operation; Recovery

**Published:** 1897-12

**Authors:** E. Mayhew Michener

**Affiliations:** North Wales, Pa.


### REPORTS OF CASES.

### INTUSSUSCEPTION OF SMALL INTESTINE OF COLT; OPERATION; RECOVERY.

By E. Mayhew Michener, V.M.D.,
NORTH WALES, PA.

On October 11, 1897, a colt, aged three months, property of Cloverdell Stock Farm, Colmar, was noticed at 6 A.M. to have colicky pains, and ordinary remedies were given without relief. I saw the colt for the first time at noon of the same day, and found it in great pain, constantly up and down, and taking the dorsal position when down. My diagnosis was some obstruction of bowels, probably intussusception or volvulus of small intestine. Prognosis, grave. Operation was accordingly advised as affording the only hope. The owner considered the matter until 4 P.M, when he decided to let me operate, and sent in great haste.

On arriving at the farm again about 6 P.M., I at once prepared and arranged an electric light, chloroformed the colt, scrubbed whole under-surface of the abdomen with hot water and soap, and sponged off with hy. bichloride, 1 : 1000 The animal was laid on a clean sheet and opened by a median incision of the abdomen seven inches in extent in front of the umbilicus. The small intestine was secured and pulled over half of its length through the opening and laid on the abdomen between the hind legs. The cause of the trouble was now revealed—an intussusception two feet three inches in length. The bowel was very much swollen and leather-like to the touch, and exceedingly weighty. By letting assistant pull steadily and carefully at the bowel while I pushed at the opposite end of the obstruction, I succeeded in righting the trouble. As the gut slipped into position there was a considerable quantity of nearly colorless liquid liberated which had transfused to the space between the two peritoneal surfaces. About one foot of the imprisoned gut was dark blue to almost black in color, and very suggestive of gangrene. After stroking the dark part with the hand for a minute or two to promote circulation, the intestines were returned to the abdomen and wound closed by three lines of sutures, row of continuous for peritoneum, one row for recti muscles and tunica abdominis, one row for skin, waxed linen thread being employed, this being the only material at hand. The abdomen was sponged with bichloride and the anæsthetic removed. Time, about twenty minutes.

The colt soon recovered from the chloroform and nursed in less than thirty minutes from finish of operation. Temperature before operation $103\frac{5}{6}$°; one hour after operation, $104\frac{1}{2}$°. The colt passed a very good night, standing most of the time, but occasionally down and resting easily. Nursed frequently and drank a small quantity of water at short intervals.

*October* 12*th*, temperature $104\frac{1}{2}$°; slight diarrhœa. Peristaltic movements energetic, causing a loud rumbling sound; otherwise doing very well.

13*th*, temperature 104°; bowels still active, but feces more solid and covered with some tough mucus. Improved in appetite and general appearance; wound doing nicely.

From this time on the temperature dropped slowly to normal. On the ninth day the stitches in the skin were removed to keep them from cutting; the wound in the skin gaped a little. Suture of recti muscles and peritoneum removed on the thirteenth day, showing complete union. There now (November 2, 1897) remains only a slight wound, rapidly healing. The animal is lively and well.

During the operation and the progress of the case I was aided by the advice and assistance of my father, Dr. J. C. Michener, of Colmar.

